# *Klebsiella pneumoniae* Lower Respiratory Tract Infection in a South African Birth Cohort: a Longitudinal Study^☆^

**DOI:** 10.1016/j.ijid.2022.04.043

**Published:** 2022-08

**Authors:** Heather J Zar, Rae MacGinty, Lesley Workman, Tiffany Burd, Gerald Smith, Landon Myer, Jonas Häggström, Mark P Nicol

**Affiliations:** a Department of Paediatrics and Child Health, Red Cross War Memorial Children's Hospital and SA-MRC Unit on Child & Adolescent Health, University of Cape Town; b Division of Epidemiology & Biostatistics, School of Public Health & Family Medicine, University of Cape Town; c Division of Medical Microbiology, University of Cape Town; d Division of Infection and Immunity, Department of Biomedical Sciences, University of Western Australia; e Cytel Inc, Sweden

**Keywords:** *Klebsiella pneumoniae*, LRTI, Hospitalization, HIV

## Abstract

**Objectives:**

The role of *Klebsiella pneumoniae* (KP) in lower respiratory tract infection (LRTI) is not well studied. We longitudinally investigated KP colonization and its association with LRTI in a South African birth cohort.

**Methods:**

We conducted a case-control study of infants who developed LRTI and age-matched controls, followed twice weekly through infancy. Nasopharyngeal swabs taken fortnightly and at LRTI for 33-multipex Quantitative multiplex real-time polymerase chain reaction were tested at LRTI and twice weekly from 90 days preceding LRTI. Controls were tested over the equivalent period. Multivariate models investigated the factors associated with LRTI or with KP-associated LRTI (KP-LRTI).

**Results:**

Among 885 infants, there were 439 LRTI episodes, of which 68 (15.5%) were KP-LRTI (OR 1.93; 95% CI 1.25–3.03). Infants with KP-LRTI were younger than those without KP-LRTI (median [IQR] 3.7 [2.1–5.9] vs 4.7 [2.8–7.9] months, *P*-value=0.009). Clinical features of KP and non-KP-LRTI were similar with 114 (26%) infants hospitalized. Prematurity (adjusted odds ratio [aOR] 11.86; 95% CI 5.22–26.93), HIV exposure (aOR 3.32; 95% CI 1.69–6.53), lower birthweight (aOR 0.68; 95% CI 0.51–0.91), and shorter breastfeeding time (aOR 0.79; 95% CI 0.65–0.96) were associated with KP-LRTI versus non-LRTI. These factors and younger age were associated with KP-LRTI versus non-KP-LRTI.

**Conclusion:**

KP was associated with a substantial proportion of LRTI, particularly in premature or HIV-exposed infants in whom strategies for treatment and prevention should be strengthened.

## Highlights

• Klebsiella pneumoniae (KP) caused a substantial proportion of lower respiratory tract infection (LRTI) in infants
• KP-LRTI was associated with prematurity, HIV exposure, or short breastfeeding time
• Preceding colonization with KP was associated with LRTI
• Premature or HIV-exposed infants with severe LRTI may require therapy for KP

## Introduction

The incidence and severity of childhood pneumonia or lower respiratory tract infection (LRTI) have declined substantially in the last decade, but LRTI remains a major cause of mortality in children under five years of age, particularly in low- and middle-income countries (LMICs) (GBD Lower Respiratory Infections Collaborators, 2018). Socioeconomic development, control of the HIV pandemic, and strengthened strategies for child health, especially conjugate vaccines (pneumococcal conjugate [PCV], and *Haemophilus influenzae type* b [Hib] vaccines), have reduced the burden of pneumonia and changed the etiological spectrum. After the widespread uptake of PCV and Hib vaccines, other bacteria and viruses have been associated with an increasing etiological fraction of pneumonia, with coinfections occurring especially in severe disease (O'Brien et al., 2019; Zar et al., 2016).

*Klebsiella pneumoniae* (KP) has been reported to be an important bacterial pathogen that causes neonatal sepsis and mortality, predominantly in southeast Asia (Downie, et al., 2013; Saha, et al., 2018). The Aetiology of Neonatal Infections in South Asia (ANISA) study, done in Bangladesh, India, and Pakistan, found that KP was an important cause of bacteremia and of mortality in neonates (Saha, et al., 2018). *K. pneumoniae* has also been reported as a cause of bacteremic pneumonia in hospitalized children in India, Thailand, and Ethiopia (Kanoksil, et al., 2013; Mathew, et al., 2015; Negash, et al., 2019). However, the role of KP in community-acquired LRTI in infants has not been well studied, especially in the context of high coverage with PCV and Hib. Cross-sectional studies predominantly of children hospitalized with pneumonia have inconsistently reported KP as a pathogen (Goyet, et al., 2014; Kurade, et al., 2018; Ning, et al., 2017), but there are no published longitudinal studies of the epidemiology, risk factors, and outcome of KP-associated LRTI. Challenges in microbiologically confirming KP may account for some of this lack of data; for example, the recent Pneumonia Etiology Research for Child Health (PERCH) cross-sectional study of children hospitalized with severe or very severe pneumonia in seven LMICs did not report the incidence of KP pneumonia (O'Brien et al., 2019). Understanding the role of KP is important because the disease may require specific antibiotic therapy, and novel strategies for prevention may be needed.

We reported a high incidence of LRTI in children in early childhood in the Drakenstein Child Health study (DCHS), a South African birth cohort study (Zar et al., 2016). The aim of this study was to longitudinally investigate the epidemiology, risk factors, and outcome of KP-LRTI from birth through infancy and the role of preceding nasopharyngeal colonization.

## Methods

### Study design and participants

We conducted a case-control study of children enrolled in the DCHS, a birth cohort study situated in a periurban area in South Africa (Zar et al., 2015). This area has a strong public primary health care program including antenatal care, prevention of maternal to child HIV transmission and immunization. Pregnant women were enrolled during their second trimester at two public sector primary healthcare clinics, TC Newman and Mbekweni. Inclusion criteria were being 18 years or older, 20–28 weeks gestation, and resident in the area. Gestational age was measured by an antenatal ultrasound done in the second trimester; if this was unavailable, then symphysis-fundal height, recorded by trained clinical staff at enrollment or maternal recall of last menstrual period was used. All births occurred at Paarl hospital, from May 29, 2012–September 3, 2015. Mother-infant pairs were followed up from birth, with study visits synchronized with routine health or immunization visits (diphtheria, tetanus, acellular pertussis, Hib, and inactivated polio vaccine at six, 10, 14 weeks; measles vaccine at 9 months; and 13-valent PCV at 6 weeks, 14 weeks, and 9 months, according to the national program). Additional study visits were done at 6 and 12 months. Mother-infant pairs were offered the option of participating in an intensive twice weekly study follow-up during the first year. Disenrollment followed at least three unsuccessful attempts by phone and home visits to locate a participant.

Longitudinal measurement of risk factors for LRTI including immunizations, smoke exposure, HIV exposure, nutrition, home environment, and maternal factors was done at study visits and during illness. Maternal smoking was self-reported antenatally and postnatally. Maternal HIV status was ascertained during pregnancy and CD4 count and viral load were measured; combination antiretroviral therapy (ART) was provided for all women infected with HIV, according to national guidelines, as described (Zar et al., 2019). Infants were tested for HIV according to national guidelines. Socioeconomic status (SES) was measured using a validated composite measure, encompassing current employment, education, household income, and an asset index.

The Revised World Health Organisation (WHO) criteria were used for pneumonia or LRTI, as described (Zar et al., 2020). Pneumonia or LRTI was diagnosed when a child had cough or difficulty breathing, with either lower chest wall indrawing or age-specific tachypnea (≥ 50 breaths per minute if 2–12 months, ≥40 breaths per minute if ≥12 months). Severe pneumonia was diagnosed if a general danger sign occurred (cyanosis, decreased level of consciousness, inability to feed, severe respiratory distress, vomiting everything, seizures) or if a child was under 2 months of age with signs of pneumonia. Active surveillance for LRTI was done at clinics and the single central hospital by the research team; mothers or healthcare personnel could also directly contact a member of the study team through a 24-hour study cell phone. Children were assessed by trained study staff at each episode of LRTI and were followed up through hospitalization or ambulatory illness. Active surveillance for LRTI was done at local clinics and at Paarl hospital, with mothers having 24 hour telephonic access to a member of the study team should their child get ill as described (Le Roux, et al., 2015).

### Microbiological investigations

Nasopharyngeal swabs (NPs, FLOQSwabs^TM^, Copan Diagnostics, CA) were collected twice weekly for the first year of life in the intensive cohort and at the time of LRTI. Nasopharyngeal swabs were transferred into nucleic acid preservation medium (PrimeStore, Longhorn Vaccines and Diagnostics, San Antonio, TX, USA), transported on ice, and frozen at –80°C for batch testing. Swabs were tested at the time of LRTI and twice weekly up to 90 days before LRTI with qPCR. Swabs from age-matched control children without LRTI in the cohort were also tested over the equivalent period. Nucleic acid was extracted using mechanical lysis on a Tissuelyzer LT (Qiagen, Germany), followed by extraction with the QIAsymphony® Virus/Bacteria mini kit (Qiagen, Germany). Quantitative multiplex real-time PCR (qPCR) was done using FTDResp33 (Fast-Track Diagnostics, Esch-sur-Alzet, Luxembourg) to identify up to 33 potential respiratory pathogens including KP. Due to previous concerns regarding false-positive results for the *K. pneumoniae* target in this assay, we did careful manual review of all amplification curves (both for samples and *K. pneumoniae* positive and negative controls, blinded to case/control status) and were able to exclude results which were likely to represent false-positive tests on the basis of (i) nonexponential amplification (nonsigmoidal amplification curve) and (ii) application of a strict cycle of quantitation (Cq) -cutoff value of <35 cycles for a positive result. In the majority of cases, likely false-positive results were clearly apparent, with weak, nonexponential amplification curves. *K. pneumoniae*-LRTI was defined as any LRTI positive for KP on qPCR.

### Ethics

The study was approved by the Faculty of Health Sciences Research Ethics Committee, University of Cape Town and Western Cape Provincial Research Committee. Mothers gave written informed consent at enrollment and reconsented annually.

### Analysis

The case-control dataset was derived from all infants included in the intensive follow-up cohort. Cases were selected on the basis of an LRTI episode in the first year of life and a valid NP available at the time of LRTI, as well as having a matched control. Controls were selected from age-matched children in the cohort who had been followed up twice weekly with NP swab collection and who had a valid NP result at the corresponding time point to enable comparison with cases*.* Controls were 1:1 matched to cases by birth date (within two weeks) and age of presentation (within two weeks). NPs were used for identification of potential pathogens to enable a case-control analysis because lower respiratory tract samples could not be collected in healthy controls. LRTI episodes were stratified into those in whom the swab collected at LRTI was positive for *K. pneumoniae* and associated with LRTI (KP-LRTI) and those in whom the swab was negative but positive for other organisms (non-KP-LRTI). Controls were similarly stratified on the basis of whether the swab was positive or negative for *K. pneumoniae* (KP controls or non-KP controls).

Prematurity was defined as birth <37 weeks gestation with late prematurity as a gestational age of 34 to 37 weeks. Weight-for-age at birth Z-scores were derived from Fenton's growth standards (Fenton and Kim, 2013), while weight-for-age Z-scores post birth were generated using the WHO child growth standards (WHO Multicenterre Growth Reference Study Group, 2006). Data were analyzed using STATA 14.1 (STATA Corporation, College Station, TX USA) and R (R Core Team, 2015). Data were summarized with frequency (percent) if categorical and median (interquartile range) if continuous. Kruskal-Wallace, Wilcoxon rank-sum test and Chi-square or Fisher's exact was used for crude comparison, as appropriate.

Analysis was done on a per child basis. We used a longitudinal analysis technique, generalized estimating equation models specifying a binomial distribution, and logit link function, which allowed for repeated measures per child because a child could have multiple LRTI episodes, or could be used as a control multiple times, and/or could switch status. Because the outcome (in Model A) was case status, this longitudinal analysis allowed for the status to change or to be repeated multiple times per child. In subsequent models (Models B and C), the case status was stratified on the basis of KP status. There was at least a 28-day period on either side of the episode between which a second event could occur. We used these models to determine risk factors associated with LRTI versus non-LRTI (Model A), KP-LRTI versus non-LRTI (Model B), or KP-LRTI versus non-KP-LRTI (Model C) at the time of event. Multivariate models were adjusted for an *a priori* set of confounding factors, including: sex, household income, parent employment status, season of birth, HIV exposure, maternal smoking, prematurity, birth weight, and age at LRTI.

## Results

**Figure 1:**
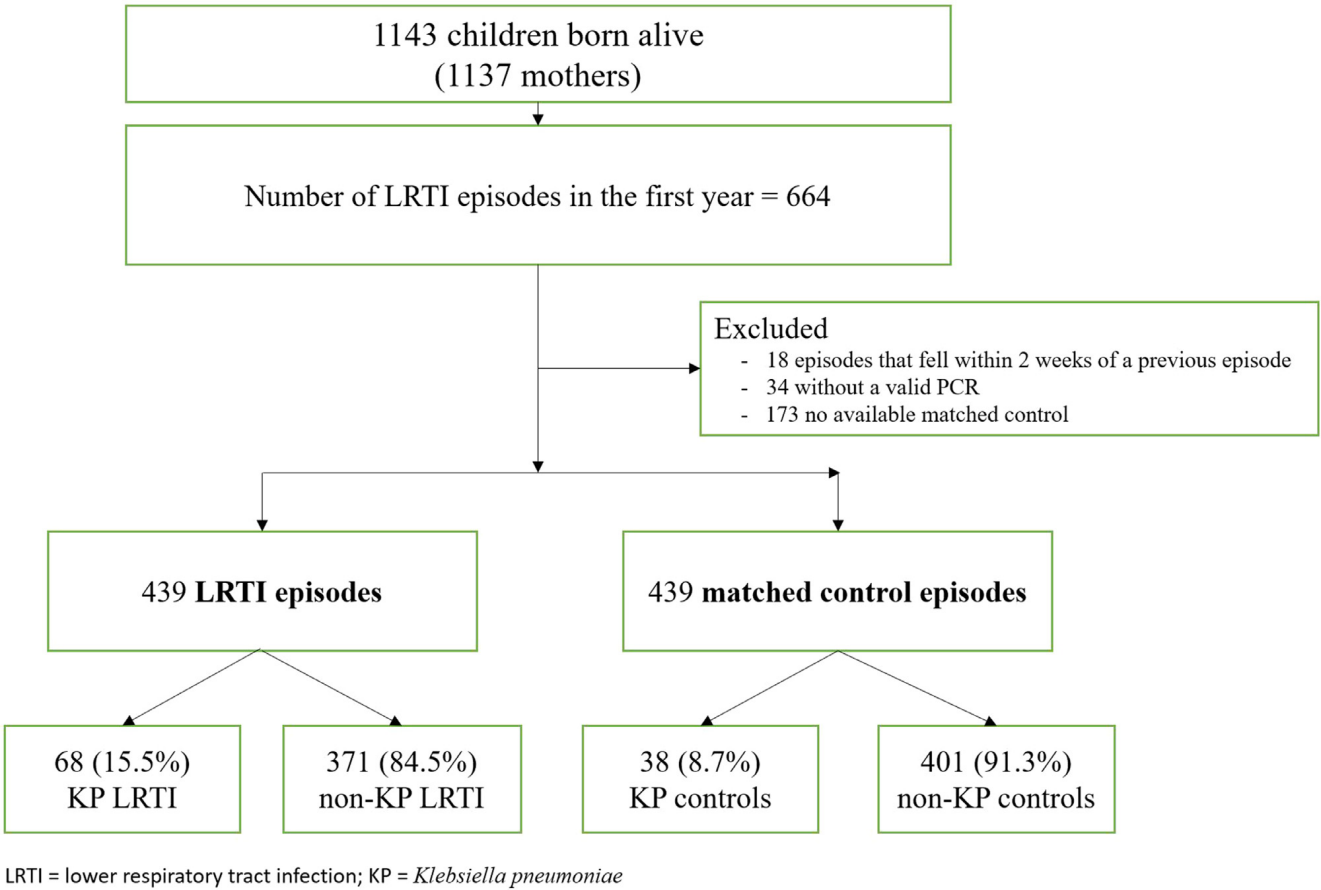
Flow diagram of cases and controls in the cohort

From March 5, 2012–March 31,2015, 1137 mothers (median [IQR] age 26 [22–31] years) were enrolled, yielding 1143 live births (four sets of twins, one set of triplets). More than three quarters of mothers (n=879, 77.3%; 885 infants) chose to participate in two-weekly follow-up; among these, cohort retention at one year was 88.7% (n=780/879) (Figure 1). Most disenrollment (n=105, 11.9%) was due to relocation (n=38, 4.3%) or inability to contact the mother (n=22, 2.5%). There were 439 LRTI episodes (among 273 infants, including recurrent LRTI episodes) with a valid PCR result on a NP sample and matched control, of which 68 (15.5%) were KP-LRTI (Figure 1). There were three infant deaths, giving an overall mortality rate of 0.57%; two were related to an LRTI, of which one was KP-LRTI.

Cases were matched 1:1 to 439 control samples from age-matched infants without LRTI. In this case-control set, there were 527 infants corresponding to 526 mothers (there was one set of twins); 228 children had an LRTI episode/s only, 254 were controls only (i.e., no LRTI episode), and 45 children were used as a case and a control at different times in the first year. Cases excluded due to the absence of the valid PCR result or age-matched control were similar to those included except for a higher prevalence of maternal smoking, Supplementary Table 1. Amongst mothers, 144 (27.4%) were HIV-infected, 99% of whom took ART in pregnancy, with most having CD4 count >250 cells/mm^3^ (Table 1). There were only 2 (0.4%) infants infected with HIV (Table 1). The population were predominantly of low socioeconomic status with the median monthly income <R5000 ($333), and 257 (48.9%) having a parent employed. Smoking was self-reported by 138 (26.2%) mothers during pregnancy and 144 (27.7%) postnatally. There were 89 (16.9%) infants born prematurely, of whom 55 (61.8%) were late preterm births. Most mothers (n=481/526, 91.4%) initiated breastfeeding but the duration of exclusive breastfeeding was short (median 1.4 [0.5–3.0] months). Immunization coverage was high with >99% coverage for six-, 10-, and 14-week vaccines and 98.8% for nine-month vaccination (Table 1).

**Table 1:** Maternal and child characteristics of participants in the matched case-control analysis

Measure	Total (n, %)
**Maternal characteristics (n=526)**	
Antenatal self-report smoking	138 (26.2)
Postnatal self-reported smoking^1^	144 (27.7)
HIV-infected	144 (27.4)
CD4 cell count (cells/ mm^3^)	
≥ 500	46/110 (41.8)
250–500	46/110 (41.8)
< 250	18/110 (16.4)
Member of household employed	257 (48.9)
Household Income category <1000 per month [ZAR]	201 (38.2)
1000–5000 per month [ZAR]	263 (50.0)
>5000 per month [ZAR]	62 (11.8)
**Birth charateristics (n=527 infants)**	
Vaginal delivery	420 (79.7)
Premature (<37 weeks gestation)	89 (16.9)
Gestational age of preterm infants [median (IQR)]	35 (32–36)
Duration of hospitalization after birth in preterm [median (IQR)]	2 (1.0–18.5)
**Child characteristics**	
Male, n (%)	281 (53.3)
Birth weight-for-age z score [median (IQR)]	–0.6 –(-1.3–0.1)
HIV-infected	2 (0.4)
HIV-exposed uninfected	142 (26.9)
Duration (months) exclusive breast feeding [median (IQR)]	1.4 (0.5–3.0)
Vaccine coverage	
6 weeks	514/515 (99.8)
10 weeks	514/515 (99.8)
14 weeks	510/512 (99.6)
9 months	477/483 (98.8)

IQR = Interquartile range; HIV = human immunodeficiency virus; ZAR= South African Rand

1 Six mothers did not have postnatal smoking status recorded

Of the 232 *K. pneumoniae* qPCR results, 116 results, which would have been initially regarded as positive, were reclassified as negative following recalibration of the Cq cutoff value (only results with Cq < 35 cycles were regarded as positive), and a further 10 results with Cq < 35 cycles were regarded as negative based on nonexponential amplification curves. Using a case-control analysis, detection of *K. pneumoniae* at the time of LRTI was significantly associated with LRTI (OR 1.93; 95% CI (1.25-3.03) (Table 2). Although the prevalence of *K. pneumoniae* in NPs at each timepoint twice weekly up to 90 days preceding LRTI was higher in those who developed LRTI than controls, this was not significant at any preceding time point (Table 2). Among LRTI cases, 114 (26.0%) required hospitalization; this was similar in KP and non-KP-LRTI, table 3. Among KP-LRTI hospitalized cases, 9/18 (50%) were treated empirically with a combination of intravenous ampicillin and gentamicin; the rest received ampicillin or high-dose amoxicillin as the microbiological results were unknown at the time of initiation of treatment. All improved and were discharged except for a single in-hospital death. In ambulatory cases, children were treated empirically with oral amoxicillin, with recovery in all. Infants with KP-LRTI were younger than those with non-KP-LRTI (3.7 vs 4.7 months), were more likely to be HIV-exposed (36 [52.9] %) vs 97 [26.2]%)], premature (32 [47.1]%) vs 75 [20.2%]), or have a lower weight-for-age z score –(-0.6 vs 0) at the time of LRTI (Table 3). Clinical features in KP and non-KP-LRTI were similar except that cough or rhinorrhea were less common in KP-LRTI (Table 3).

**Table 2:** Odds ratio of *K. pneumoniae* at time of LRTI and at two-weekly intervals preceding LRTI in cases vs controls

	Time of LRTI	1–14 days prior to LRTI	15–28 days prior to LRTI	29–56 days prior to LRTI	>57 days prior to LRTI	Overall
	OR (95% CI)	OR (95% CI)	OR (95% CI)	OR (95% CI)	OR (95% CI)	OR (95% CI)
Case vs Control	**1.93** **(1.25–3.03)**	1.09 (0.62–1.94)	1.48 (0.87–2.52)	1.01 (0.70–1.45)	1.30 (0.89–1.90)	1.30 (1.07–1.56)

LRTI = lower respiratory tract infection

**Table 3:** Features of *K. pneumoniae*-LRTI compared with non-KP-LRTI in infants

	All LRTIn (%)	KP-LRTIn (%)	Non-KP-LRTIn (%)	OR (95% CI)
n (%)	439 (100)	68 (15.5)	371 (84.5)	
Median (IQR) age at LRTI	4.6 (2.7-7.5)	3.7 (2.1–5.9)	4.7 (2.8–7.9)	**0.87 (0.79**–**0.95)**
Preterm	107 (24.4)	32 (47.1)	75 (20.2)	**3.54 (1.95**–**6.41)**
Median (IQR) gestational age at birth	39 (37–40)	37 (33–39)	39 (37–40)	**0.98 (0.97**–**0.99)**
Weight-for-age z score at LRTI	–0.2 (-1.3–0.9)	–0.6 (-2.9–0.2)	–0.0 (-1.2–1.0)	**0.75 (0.64**–**0.87)**
HIV-exposed	133 (30.3)	36 (52.9)	97 (26.2)	**3.01 (1.70**–**5.36)**
Season of LRTI				
Summer (Dec–Feb)	70 (16.0)	14 (20.6)	56 (15.1)	reference
Autumn (Mar–May)	117 (26.7)	22 (32.4)	95 (25.6)	0.89 (0.43–1.82)
Winter (Jun–Aug)	121 (27.6)	16 (23.5)	105 (28.3)	0.55 (0.26–1.16)
Spring (Sep–Nov)	131 (29.8)	16 (23.5)	115 (31.0)	0.54 (0.25–1.14)
**Symptoms**				
Fever^1^	271 (62.9)	44 (67.7)	227 (62.0)	1.19 (0.69–2∙07)
Cough	418 (95.2)	59 (86.8)	359 (96.8)	**0.22 (0.09**–**0.53)**
Rhinorrhoea	224 (51.0)	17 (25.0)	207 (55.8)	**0.28 (0.16**–**0.51)**
Vomiting^2^	51 (12.0)	5 (7.8)	46 (12.8)	0.60 (0.24–1.51)
Diarrhoea^3^	51 (12.0)	7 (11.1)	44 (12.2)	0.97 (0.43–2.19)
**Clinical signs**				
Median (IQR) Heart rate	143 (134–163)	141 (133–164)	144 (134–163)	0.99 (0.98–1.01)
Median (IQR) Respiratory rate	58 (52–63)	60 (52–68)	57 (52–62)	1.02 (0.99–1.05)
Lower chest wall indrawing	325 (74.2)	56 (82.4)	269 (72.7)	1.69 (0.87–3.28)
Chest auscultation abnormal^4^	245 (57.0)	34 (50.8)	211 (58.1)	0.74 (0.44–1.24)
Wheezing	162 (36.9)	23 (33.8)	139 (37.5)	0.89 (0.52–1.52)
Median (IQR) O_2_ saturation at LRTI	97 (95–98)	97 (94–98)	97 (95–98)	0.98 (0.94–1.02)
Hospitalized	114 (26.0)	18 (26.5)	96 (25.9)	1.00 (0.56–1.78)

LRTI = lower respiratory tract infection; OR = odds ratio for comparison of KP-LRTI vs non-KP-LRTI; 95% CI = 95% confidence interval KP = *Klebsiella pneumoniae*; HIV = human immunodeficiency virus; IQR= interquatile range

1 8 participants missing response (3 from KP-LRTI group, 5 from non-KP-LRTI group)

2 15 participants missing response (4 from KP-LRTI group, 11 from non-KP-LRTI group)

3 15 participants missing response (5 from KP-LRTI group, 10 from non-KP-LRTI group)

4 9 participants missing response (1 from KP-LRTI group, 8 from non-KP-LRTI group)

Bolded values indicate significant results

Multivariate modeling of risk factors associated with all-cause LRTI included male sex (aOR=1.47, 95% CI 1.04–2.07), prematurity (aOR=2.32, 95% CI 1.42–3.78), or lower birth weight (aOR 0.84, 95% CI 0.72–0.99) (Table 4). For KP-LRTI, prematurity, HIV exposure, or lower birth weight were strong risk factors compared with infants without LRTI or those who had non-KP-LRTI (Table 4). In addition, younger age at LRTI or shorter duration of breastfeeding were risk factors for KP-LRTI compared with non-KP-LRTI (Table 4). Among infants exposed to HIV, prematurity was also strongly associated with KP-LRTI, whereas longer duration of exclusive breast feeding and higher household income was protective, but maternal antenatal CD4 count was not associated (Supplementary Table 2). Quantitative KP load in all NPs was higher in infants exposed to HIV than those who were unexposed when considering all time points, but there was no difference in bacterial load at the time of LRTI among KP-LRTI cases by HIV exposure (Supplementary Figure 1). We explored multivariate models with an interaction term between HIV exposure and prematurity, but the interaction was not significant; prematurity and HIV exposure remained strongly associated with LRTI or KP-LRTI with inclusion of the interaction term (Supplementary Table 3).

**Table 4:** Results of multivariate modeling of risk factors associated with any LRTI (model A), KP-LRTI versus no LRTI (model B) or KP-LRTI among LRTI cases (model C)

	Model A:All LRTI vs all controls		Model B: KP-LRTI vs no LRTI	Model C: KP-LRTI vs non-KP-LRTI
	Unadjusted OR(95% CI)	Adjusted* OR(95% CI)	Adjusted* OR(95% CI)	Adjusted* OR(95% CI)
**Child characteristics**				
Sex: Male (ref. female)	**1.53 (1.10–2.12)**	**1.47 (1.04–2.07)**	1.55 (0.77–3.10)	1.56 (0.79–3.07)
Preterm (<37 weeks gestation)	1.96 **(**1.26–3.03**)**	**2.32 (1.42–3.78)**	**11.86 (5.22–26.93)**	**6.85 (3.24–14.49)**
Birth weight-for-age z score	0.91 (0.79–1.05)	**0.84 (0.72–0.99)**	**0.68 (0.51–0.91)**	**0.69 (0.51–0.93)**
HIV-exposed	1.40 (0.97–2.02)	1.33 (0.90–1.96)	**3.32 (1.69–6.53)**	**2.92 (1.52–5.59)**
Season of birth (ref. summer)				
Autumn (Mar–May)	1.20 (0.77–1.89)	1.23 (0.77–1.96)	0.63 (0.25–1.59)	0.71 (0.30–1.66)
Winter (Jun–Aug)	1.04 (0.66–1.63)	1.05 (0.66–1.68)	0.90 (0.37–2.19)	0.78 (0.33–1.84)
Spring (Sep–Nov)	1.10 (0.69–1.76)	1.13 (0.70–1.84)	1.00 (0.40–2.51)	0.69 (0.28–1.72)
Age at LRTI	1.01 (0.98–1.04)	1.01 (0.98–1.05)	0.92 (0.84–1.02)	**0.90 (0.81–0.99)**
**Maternal characteristics**				
Antenatal self-reported smoking	0.91 (0.63–1.32)	0.74 (0.32–1.72)	0.21 (0.04–1.05)	0.30 (0.08–1.13)
Postnatal self-reported smoking	1.00 (0.69–1.44)	1.31 (0.58–2.99)	3.70 (0.83–16.50)	1.54 (0.46–5.13)
Household income per month (ref <1000 [ZAR])				
100–5000 [ZAR]	0.88 (0.62–1.25)	0.87 (0.60–1.28)	0.54 (0.26–1.11)	0.66 (0.32–1.38)
>5000 [ZAR]	1.01 (0.59–1.73)	0.94 (0.52–1.71)	0.74 (0.22–2.44)	0.64 (0.19–2.15)
Employment	1.05 (0.76–1.46)	1.19 (0.83–1.72)	1.22 (0.60–2.48)	1.03 (0.51–2.07)
Duration exclusive breast feeding (months)	0.96 (0.88–1.04)	0.96 (0.88–1.05)	**0.79 (0.65–0.96)**	**0.78 (0.64–0.96)**

LRTI = lower respiratory tract infection; OR = odds ratio; 95% CI = 95% confidence interval; KP = *Klebsiella pneumoniae*; HIV = human immunodeficiency virus; ZAR= South African Rand

⁎ Adjusted for all covariates displayed in table Bolded values indicate significant results

There was a higher incidence of RSV, influenza, or parainfluenza virus detected in infants with all-cause LRTI than those without LRTI but no differences in other viruses or bacteria. However, the odds of detecting *S. pneumoniae* were lower among infants with KP-LRTI than those with non-KP-LRTI adjusted for age, but there were no other differences in coinfections (Table 5).

**Table 5:** Results of multivariate modeling of other organisms associated with any LRTI (model A) or with *K. pneumoniae*-LRTI cases (model B)

	Model A: All LRTI vs all controls	Model B: KP-LRTI vs non-KP-LRTI
	Adjusted OR (95% CI)	Adjusted OR (95% CI)
***Viruses***		
*RSV*	**3.00 (2.06–4.36)**	0.86 (0.42–1.77)
*Influenza* A,B	**2.80 (1.22–6.41)**	1.20 (0.30–4.84)
*Parainfluenza*	**1.75 (1.10–2.77)**	1.01 (0.41–2.48)
*Adenovirus*	1.38 (0.95–2.00)	0.85 (0.33–2.19)
*Metapneumovirus*	1.39 (0.91–2.14)	1.34 (0.57–3.19)
*Rhinovirus*	0.96 (0.75–1.21)	1.37 (0.78–2.40)
*Cytomegalovirus*	0.96 (0.75–1.23)	0.91 (0.51–1.62)
***Bacteria***		
*S aureus*	1.08 (0.81–1.43)	0.98 (0.52–1.85)
*S pneumoniae*	1.17 (0.93–1.47)	**0.55 (0.32–0.94)**
*M catarrhalis*	1.16 (0.90–1.50)	0.78 (0.44–1.39)
*H influenzae (nontypeable)*	1.21 (0.95–1.55)	0.71 (0.40–1.26)

LRTI = lower respiratory tract infection; OR = odds ratio; 95% CI = 95% confidence interval; KP = *Klebsiella pneumoniae*

Models adjusted for age at episode/reference

Bolded values indicate significant results

## Discussion

This birth cohort study found that *K. pneumoniae* was associated with community-acquired LRTI in African infants, especially in those who had been born prematurely or who were HIV-exposed. The finding that KP was associated with LRTI in the context of a well-immunized infant population, including Hib and PCV13, almost no HIV infection in children and good nutrition is novel and important, with possible consequences for empiric treatment. Further, KP-LRTI occurred beyond the neonatal period, at a median age of 3.7 months. KP has previously been described as an important cause of neonatal sepsis or nosocomial pneumonia (Downie et al., 2013; Saha et al., 2018). The Child Health and Mortality Prevention Surveillance (CHAMPS) study, done in seven countries in South Asia and sub-Saharan Africa, investigating neonatal and childhood deaths using minimally invasive tissue sampling, reported KP as contributing to death in 31% of 275 child deaths with infectious causes (Taylor et al., 2020). A retrospective cross-sectional study of 410 South African children hospitalized with KP-bacteremia found that 27% died within 30 days of infection (Buys et al., 2016). Our data provide new information on the epidemiology of KP-LRTI in infants and on specific risk factors; reassuringly, the outcomes were favorable with around a quarter requiring hospitalization and a single death associated with KP-LRTI.

Although it may be difficult to distinguish causality from colonization, through the use of a case-control design and with controls age-matched and from the same cohort, we showed that *K. pneumoniae* was significantly associated with LRTI. The multicentre PERCH study also used a case-control methodology to attribute etiology from NP sampling of children hospitalized with severe or very severe pneumonia using the same PCR platform (O'Brien et al., 2019). Although PERCH was unable to report on KP-LRTI owing to technical challenges with this measurement, we were able to optimize the FTDResp33 methodology, as described, to enable valid KP results. Although culture may have provided results on antimicrobial susceptibility of bacteria, PCR was used because this enabled testing for multiple copathogens (including viruses) and was consistent with the methodology used in PERCH (O'Brien et al., 2019). More definitive sampling, such as a lung aspirate or bronchoalveolar lavage, is not feasible in such community-based studies, and no child had bacteremic disease. A recent systematic review reported that detection of KP colonizing the upper respiratory tract of infants was associated with LRTI (Claassen-Weitz, et al., 2021). Further, longitudinal quantitative PCR measurements at the time of LRTI and twice weekly up to 90 days preceding LRTI, from birth through infancy, enabled us to carefully investigate the epidemiology and role of preceding colonization in KP-LRTI. Although there was a higher prevalence of KP in preceding samples at all time points in those who developed KP-LRTI, this was not significantly higher than in those who did not develop LRTI. However, lack of power to detect significant changes may be due to the sample size, and preceding colonization of the nasopharynx may underlie susceptibility to KP-LRTI (Claassen-Weitz, et al., 2021).

Prematurity was the strongest risk factor for KP-LRTI despite that most premature infants being born after 34 weeks and not requiring admission to the ICU. This highlights a group of infants that have not been previously recognized to be at increased vulnerability to KP disease but who constitute around 11% of births worldwide (Chawanpaiboon, et al., 2019). Mechanisms for this vulnerability may include the lack of transplacental transfer of protective antibodies that occurs especially during the last weeks of gestation, lack of breastfeeding, or a relatively immature immune system. HIV exposure was also an important risk factor for KP-LRTI despite almost all mothers infected with HIV being on combination ART through pregnancy, with most virally suppressed and immune reconstituted (Pellowski, et al., 2019). As PMTCT programs are strengthened, the number of perinatally infected children with HIV has decreased with a concomitant increase in numbers of HIV-exposed uninfected (HEU) infants (Slogrove, et al., 2020). We have previously reported that HEU infants have an increased risk of LRTI especially in the first six months of life (Le Roux, et al., 2019); herein, we extend this work by demonstrating that they have an increased risk of KP-LRTI compared with HIV-unexposed infants. This vulnerability to KP may be due to lack of protective antibodies, lack of breastfeeding, higher rates of KP exposure from a mother who was infected with HIV, or a specific immune defect. Further higher KP bacterial load, as occurred in all preceding NP samples of infants who were HIV-exposed compared with those unexposed, may be important in the pathogenesis of disease. Duration of exclusive breastfeeding is well described as protective for LRTI generally (McAllister, et al., 2019), as we found for KP-LRTI specifically. Further study of potential underlying mechanisms for development of KP-LRTI is needed.

Although other causes of LRTI such as RSV predominated, KP-LRTI was an important, treatable cause of community-acquired LRTI. KP-LRTI occurred at a median of 3.7 months, when empiric antibiotic therapy is oral amoxicillin or intravenous ampicillin in those who cannot tolerate oral therapy as recommended by WHO and other guidelines (World Health Organization, 2014). This recommendation does not provide adequate antibiotic coverage for infants with severe KP-LRTI. However, all except one of the children with KP-LRTI in our cohort recovered despite the majority receiving antibiotic therapy without activity against *K. pneumoniae.* Copathogens or interaction with other organisms, as has increasingly been recognized in pneumonia pathogenesis, may have contributed to development of LRTI and recovery in the absence of specific treatment; however, we did not identify any specific pattern of co-pathogen detection with KP on multiplex PCR testing. Further studies are needed to determine whether *K. pneumoniae* is a significant pathogen and contributes to poorer outcomes in HIV-exposed or premature infants who require hospitalization for LRTI in other settings. The CHAMPS data similarly indicate that a high proportion of infants have *K. pneumoniae* as a contributory cause of death (Taylor, et al., 2020), adding further evidence to the need for empiric antibiotic therapy for this pathogen in specific contexts.

The reduced age-adjusted odds of detecting *S. pneumoniae* in children with KP-LRTI, compared with those with non-KP-LRTI suggests the possibility of an interaction between these two species. Both species possess an anionic antiphagocytic capsule, which may plausibly mediate contact-dependent inhibition. Commensal oral streptococcal species have been shown to have bacteriostatic activity against *K. pneumoniae;*(Li, et al., 2019), although, to our knowledge, such activity has not been demonstrated by *S. pneumoniae*.

Limitations include a relatively small sample of infants with KP-LRTI. However, this study is the only longitudinal study of KP-LRTI through infancy that carefully delineated that occurrence occurred at a median age of 3.7 months, which was younger than other causes of LRTI. A further limitation is follow-up from birth for only one year. However, the highest incidence of LRTI occurs in the first year of life, as we have previously described (Zar et al., 2020). A further limitation is attributing causality on the basis of a case-control approach using NP samples. Although sampling from the lungs would have provided a better sample, this was not operationally feasible or clinically indicated; the methodology used is similar to that used in the PERCH study, enabling comparison with other global data (Pneumonia Etiology Research for Child Health (PERCH) Study Group, 2019). These are mitigated by several strengths of this study. Besides longitudinal study of the epidemiology and characteristics of illness, careful follow-up, with high cohort retention, and active ascertainment of LRTI episodes illness make these data unique, especially in LMICs. These results may not be generalizable to LMICs where coverage for childhood immunizations, particularly Hib or PCV, may be lower or where such vaccines are not available. However, the uptake of Hib and PCV worldwide has increased (International Vaccine Access Center (IVAC), 2020), so these results may be broadly applicable in LMICs.

In summary, this study provides important new information on the association of KP with LRTI and specific risk factors in infants. These data have important implications for understanding possible organism interactions in the etiology of LRTI in infants in the context of high immunization coverage for PCV and Hib and for antibiotic therapy. In premature or HIV-exposed infants with LRTI, antibiotic therapy with coverage for *K. pneumoniae* may be considered. Strengthened strategies to reduce the risk of prematurity and promote longer exclusive breastfeeding are needed. Further study of the impact of KP-associated LRTI on long-term health in children is warranted to investigate long-term morbidity from early life illness.

## Appendix Supplementary materials

Image, application 1
