# Supplementary material for: Klebsiella pneumoniae Lower Respiratory Tract Infection in a South African Birth Cohort: a Longitudinal Study
Source: Int J Infect Dis. 2022 Aug;121:31–8. doi: 10.1016/j.ijid.2022.04.043 (PMC9174060; doi:10.1016/j.ijid.2022.04.043)
Supplement: Supplementary file 1 [file mmc1.docx]

**Supplemental figs and tables**

Supplemental fig 1. Quantitative K pneumoniae (KP) load (median, interquartile range) in HIV-exposed and unexposed infants in all preceding KP-positive nasopharyngeal samples (panel A) and at the time of LRTI (panel B).


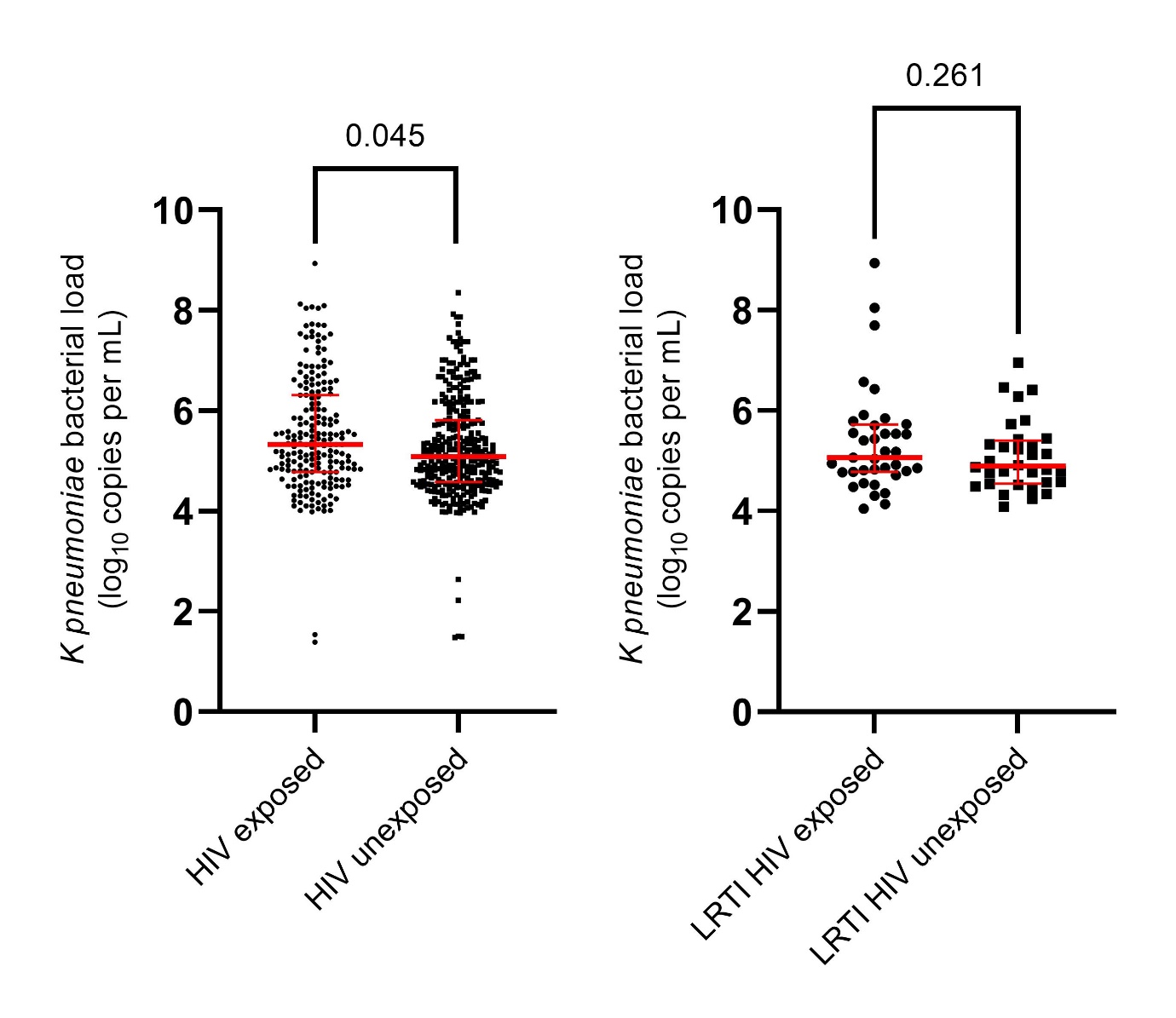


**Supplement Table 1.** Comparison of included and excluded LRTI episodes

|  | Total LRTI episodes (n=664) | Included  (n=439) | Excluded  (n=225) | OR  (95% CI) |
| --- | --- | --- | --- | --- |
| **Maternal characteristics** | | | | |
| Antenatal maternal smoking | 187 (28.2%) | 111 (25.2%) | 76 (33.8%) | **0.48 (0.26- 0.90)** |
| Household income | | | | |
| <1000 /month [ZAR] | 266 (40.1%) | 183 (41.7%) | 83 (36.9%) | Reference |
| 1000-5000 /month [ZAR] | 326 (49.1%) | 203 (46.2%) | 123 (54.7%) | 0.71 (0.39- 1.28) |
| >5000 /month [ZAR] | 72 (10.8%) | 53 (12.1%) | 19 (8.4%) | 1.56 (0.59- 4.16) |
| **Birth characteristics** | | | | |
| Vaginal delivery | 519 (78.2%) | 353 (80.4%) | 166 (73.8%) | 1.72 (0.86- 3.38) |
| Premature (<37 weeks gestation) | 170 (25.6%) | 109 (24.8%) | 61 (27.1%) | 0.73 (0.38- 1.40) |
| Gestational age [median (IQR)] | 39 (36-40) | 39 (37- 40) | 39 (36- 40) | 1.04 (0.97- 1.13) |
| **Child characteristics** | | | | |
| Male, n (%) | 407 (61.3%) | 266 (60.6%) | 141 (62.7%) | 0.81 (0.45-1.44) |
| Birth wt-for-age z score [median (IQR)] | -0.68 (-1.37- -0.01) | -0.72 (-1.35- 0.01) | -0.63 (-1.43- -0.01) | 1.09 (0.85- 1.40) |
| HIV exposed | 198 (29.8%) | 133 (30.3%) | 65 (28.9%) | 1.23 (0.66- 2.28) |
| Duration (months) exclusive breast feeding [median (IQR)] | 1.38 (0.46- 3.00) | 1.15 (0.46- 3.00) | 1.66 (0.92- 3.22) | 0.90 (0.77- 1.05) |
| Age of LRTI (months) [median (IQR)] | 4.58 (2.32- 7.69) | 4.60 (2.66- 7.43) | 4.30 (1.94- 8.44) | 1.00 (0.94- 1.07) |

OR= Odds ratio for comparison of included vs excluded; IQR = Interquartile range; HIV = human immunodeficiency virus; ZAR= South African Rand; LRTI= lower respiratory tract infection

Bolded values indicate significant results

**Supplement Table 2** Results of multivariate modelling of risk factors associated with *K. pneumoniae* associated LRTI in HIV exposed children

|  | **Model A:**  **KP-LRTI vs controls** | **Model B:**  **KP-LRTI vs non-KP-LRTI** |
| --- | --- | --- |
|  | Adjusted* OR  (95% CI) | Adjusted* OR  (95% CI) |
| **Maternal HIV measures** |  |  |
| CD4 count (cells/mm^3;^ >500 ref) |  |  |
| 250-500 | 0.68 (0.16– 2.88) | 2.52 (0.63– 10.14) |
| < 250 | 0.38 (0.05– 3.02) | 0.91 (0.16– 5.16) |
| **Child characteristics** |  |  |
| Sex: Male (ref. female) | 4.33 (0.85– 21.98) | 3.66 (0.84– 15.88) |
| Preterm | **14.95 (2.12**– **105.68)** | **41.76 (5.79– 300.91)** |
| Birth weight-for-age z score | 0.63 (0.31– 1.29) | **0.53 (0.30– 0.93)** |
| Season of birth (ref. summer) |  |  |
| Autumn (Mar-May) | 0.59 (0.09– 4.00) | 0.95 (0.14– 6.24) |
| Winter (Jun – Aug) | 0.93 (0.16– 5.34) | 0.38 (0.08– 1.78) |
| Spring (Sep – Nov) | 0.18 (0.02– 1.68) | **0.10 (0.02– 0.61)** |
| Age | 0.99 (0.84– 1.16) | 0.86 (0.70– 1.07) |
| **Maternal characteristics** |  |  |
| Antenatal Smoking | 0.35 (0.004– 28.67) | 1.02 (0.05– 19.91) |
| Postnatal smoking | 4.73 (0.08– 270.61) | 5.35 (0.46– 62.33) |
| Household income per month (ref<1000 [ZAR]) |  |  |
| 1000 – 5000 [ZAR] | **0.16 (0.03**– **0.84)** | **0.15 (0.03–0.77)** |
| >5000 [ZAR] | 0.09 (0.005– 1.51) | **0.01 (0.000- 0.36)** |
| Employment | 1.84 (0.37– 9.26) | 1.73 (0.40– 7.48) |
| Duration exclusive breast feeding (months) | **0.58 (0.37– 0.91)** | **0.40 (0.19– 0.82)** |

HIV = human immunodeficiency virus; LRTI = lower respiratory tract infection; OR = Odds ratio; 95% CI = 95% Confidence interval; K. pneumoniae (KP) = *Klebsiella pneumoniae*

*Adjusted for all covariates displayed in table

Bolded values indicate significant results

**Supplement Table 3.** Multivariate modelling of risk factors associated with all-cause LRTI (model A), KP-LRTI (model B) or KP-LRTI vs non-KP-LRTI (model C) including an interaction between preterm birth and HIV exposure.

|  | **Model A: All LRTI vs all controls** | **Model B: KP LRTI vs no-LRTI** | **Model C: KP-LRTI vs non-KP LRTI** |
| --- | --- | --- | --- |
|  | Adjusted* OR  (95% CI) | Adjusted* OR  (95% CI) | Adjusted* OR  (95% CI) |
| **Child characteristics** |  |  |  |
| Sex: Male (ref. female) | **1.46 (1.04- 2.06)** | 1.59 (0.79- 3.18) | 1.56 (0.79- 3.08) |
| Birth weight-for-age z score | **0.84 (0.72- 0.99)** | **0.69 (0.51- 0.92)** | **0.69 (0.52- 0.93)** |
| Preterm (<37 weeks gestation) | **2.26 (1.30- 3.95)** | **15.14 (5.57- 41.18)** | **6.52 (2.57- 16.55)** |
| HIV exposed | 1.31 (0.86- 1.99) | **4.09 (1.78- 9.41)** | **2.78 (1.19- 6.51)** |
| Preterm & HIV exposed^#^ | 1.09 (0.39- 3.04) | 0.54 (0.13- 2.19) | 1.13 (0.30- 4.30) |
| Season of birth (ref. summer) | | | |
| Autumn (Mar-May) | 1.23 (0.77- 1.96) | 0.64 (0.26- 1.59) | 0.70 (0.30- 1.65) |
| Winter (Jun – Aug) | 1.05 (0.66- 1.68) | 0.86 (0.35- 2.10) | 0.78 (0.33- 1.85) |
| Spring (Sep – Nov) | 1.13 (0.70- 1.84) | 0.99 (0.39- 2.48) | 0.69 (0.28- 1.71) |
| Age | 1.01 (0.98- 1.05) | 0.92 (0.84- 1.02) | **0.90 (0.81-0.99)** |
| **Maternal characteristics** | | | |
| Antenatal smoking | 0.74 (0.32- 1.71) | 0.20 (0.04- 1.02) | 0.30 (0.08- 1.13) |
| Postnatal smoking | 1.31 (0.58- 2.99) | 3.89 (0.84- 17.87) | 1.57 (0.46- 5.33) |
| Household income per month (ref<1000 [ZAR]) | | | |
| 1000 – 5000 [ZAR] | 0.87 (0.60- 1.28) | 0.55 (0.27- 1.14) | 0.66 (0.32- 1.38) |
| >5000 [ZAR] | 0.95 (0.52- 1.71) | 0.73 (0.22- 2.40) | 0.64 (0.19- 2.14) |
| Employment | 1.19 (0.83- 1.72) | 1.21 (0.60- 2.47) | 1.02 (0.51- 2.06) |
| Duration exclusive breast feeding months) | 0.96 (0.88- 1.05) | **0.79 (0.66- 0.96)** | **0.78 (0.64- 0.96)** |

LRTI = lower respiratory tract infection; OR = odds ratio; 95% CI = 95% confidence interval; KP = *Klebsiella pneumoniae*; HIV = human immunodeficiency virus

*Adjusted for all covariates displayed in table

^#^ interaction term between preterm birth and HIV exposure

Bolded values indicate significant results
